# The miR-143/miR-145 cluster and the tumor microenvironment: unexpected roles

**DOI:** 10.1186/s13073-016-0284-1

**Published:** 2016-03-17

**Authors:** Maria Inês Almeida, George Adrian Calin

**Affiliations:** Instituto de Investigação e Inovação em Saúde/Institute for Research and Innovation in Health (I3S), University of Porto, 4200-135 Porto, Portugal; Instituto de Engenharia Biomédica (INEB), University of Porto, 4200-135 Porto, Portugal; Department of Experimental Therapeutics, The University of Texas MD Anderson Cancer Center, Houston, TX 77030 USA; Center for RNAi and Non-Coding RNA, The University of Texas MD Anderson Cancer Center, Houston, TX 77030 USA

## Abstract

miR-143 and miR-145 have been widely described as tumor suppressors. Recent findings suggest these microRNAs (miRNAs) have a critical role that affects the stroma rather than epithelial cells. Understanding the part played by the miR-143/miR-145 cluster in the tumor microenvironment is essential for the development of future miRNA-related therapies.

## Tumor suppression

The contribution of the microenvironment to promoting tumor growth has been recognized for decades [1]. Immune cells, fibroblasts, endothelial cells, mesenchymal stem cells, pericytes and, in some cases, adipocytes, are among the cell types that surround and interact with tumor cells, forming the tumor microenvironment [1]. A tumor generates a local response that leads to modifications of the extracellular matrix at the molecular, biological and mechanical levels, which in turn supports tumor growth and migration [1]. The surrounding cells secrete a cocktail of tumor-promoting factors, including cytokines, chemokines, pro-angiogenic and anti-inflammatory mediators, and extracellular vesicles, which all play a part in stimulating tumor progression [1]. Therefore, the cells present in the tumor stroma should be considered when studying the underlying biological mechanisms and therapeutic approaches for epithelial tumors.

MicroRNAs (miRNAs) are small non-coding RNAs that regulate gene expression at a post-transcriptional level, inhibiting mRNA translation or degrading mRNA. They are crucial players in tumorigenesis, and can act as oncogenes or tumor suppressors [2]. One set of miRNAs that has been intensively studied is the miR-143/145 cluster, which is formed by two co-transcribed but distinct miRNAs. This cluster has been described as having tumor suppressive functions in several tumor types that have an epithelial origin, such as cervical, colon, gastric, breast and pancreatic carcinomas [3]. This has been shown by miRNA expression analyses in tumor and normal tissue samples and in gain-of-function and loss-of-function studies, both in vitro and in vivo [3]. However, a recent well-designed study performed by Dimitrova, Gocheva and colleagues contradicts the concept of the miR-143/miR-145 cluster as a classical tumor suppressor [4]. miR-143 and miR-145 have been considered ideal candidates for cancer therapy, and several strategies for delivery of these miRNAs into cells are being explored [3]. Therefore, this study has important implications.

## Tumor promotion

To explore the endogenous role of the miR-143/miR-145 cluster, Dimitrova, Gocheva and colleagues initially used mice with a conditional knockout for miR-143/miR-145, but these animals failed to develop tumors, and differences in survival between these mice and controls were not detected [4]. Next, the researchers induced tumor-specific deletion of miR-143/miR-145 in a lung cancer mouse model (Kras^G12D/+^, p53^–/–^) in which tumors resemble human lung tumors. However, no differences in tumor burden or survival rate were found. In the same model, mice infected with a conditional lentivirus vector for forced expression of either miR-143 or miR-145 showed no difference in tumor burden when compared with controls. These results contradict earlier suggestions that the miR-143/miR-145 cluster has a tumor suppressor role [4]. Surprisingly, in the lung cancer mouse model, animals with a miR-143/miR-145 knockout (an organism-wide deletion) showed decreased number of tumors and reduced tumor area compared with controls. The same effect was also present after mice tail vein injection of Kras^G12D/+^, p53^–/–^ cells with miR-143/miR-145 null alleles. Altogether, these results suggested that systemic miR-143/miR-145 expression could support tumor growth and launched the hypothesis that these miRNAs affected the stroma rather than epithelial cells [4].

To further explore the role of the tumor microenvironment, stromal cells were isolated and sorted, and the researchers found that endothelial cells, but not epithelial cells, were enriched in miR-143/miR-145. In miR-143/miR-145-deficient mice, active neoangiogenesis was reduced, and this effect could be partially explained by miRNA targeting of calcium/calmodulin dependent protein kinase ID (Camk1d), a protein involved in angiogenesis [4]. Specifically, miR-145 binds to the 3’ untranslated region (UTR) of Camk1d and represses its expression. This study points to the miR-143/miR-145 cluster as a critical promoter of neoangiogenesis [4]. This finding is of crucial importance, as gain-of-function therapeutic strategies using this cluster could promote neoangiogenesis in the lung cancer microenvironment and support tumor growth instead of suppressing it.

This is the second in vivo study arguing against a tumor-suppressor function for the miR-143/miR-145 cluster [5]. Although exogenous forced-expression studies had suggested that the miR-143/miR-145 cluster is a tumor suppressor in colorectal cancer, Chivukula, Shi and collaborators studied the role of these miRNAs in intestinal physiology and failed to detect abnormalities in the development or architecture of the small and large intestine in miR-143/miR-145-deficient mice [5]. However, these mice could not recover from intestinal injury. Moreover, these researchers detected miR-143 and miR-145 in mesenchymal cells but not in epithelial cells of the intestine [5]. Together, these results suggests that the stromal microenvironment is regulated by these miRNAs rather than the intestinal epithelium. Depletion of miR-143 and miR-145 caused dysfunction of smooth muscle cells and myofibroblasts, which in turn impaired the regeneration of the intestinal epithelium. This effect was explained, at least in part, by miR-143 targeting of insulin-like growth factor binding protein 5 (IGFBP5), which in turn negatively regulates the insulin-like growth factor signaling pathway, known to be critical for intestinal epithelial proliferation [5].

In the majority of gene expression studies in cancer, RNA is isolated from heterogeneous tumor samples. This approach can generate misleading conclusions, as expression of a miRNA exclusively in one specific cell type can appear as bulk expression [6]. Therefore, when studying gene expression levels in epithelial tumor cells, the stromal bias should be considered. Analysis of gene expression levels after fluorescence-activated cell sorting for distinct cell populations could overcome this issue [4]. Another approach could be the use of laser capture microdissection, which enables single cell isolation based on morphology or presence or absence of cell markers [7]. Finally, in situ hybridization could also be used to determine expression of miRNAs in specific cell types and their location within the tissue sample [8].

Delivery of exogenous miRNAs is considered as one of the most promising novel strategies for treatment of human cancer [9]. However, it is important to consider the endogenous expression and the effects of miRNAs in vivo on the tumor microenvironment, as elegantly revealed by Dimitrova, Gocheva and collaborators [4]. Therefore, care should be taken when selecting miRNAs for exogenous therapeutic delivery. A direct-targeting system specific for tumor cells is essential to avoid exposure of the tumor microenvironment to treatment. Some miRNAs might cause apoptosis and reduce proliferation of tumor epithelial cells but at the same time increase pro-tumorigenic mediators secreted by stromal cells. Moreover, the number and type of stromal cells present in the tumor microenvironment might differ in respect to the tumor type. Therefore, the expression and effects of miRNAs in the stroma should be dissected in every type of tumor and the potential of miRNA communication through miRNA secretion in the microenvironment should be tested. This type of communication was shown, for example, for miR-21, which is secreted by epithelial malignant cells and induces the release of miR-155 by monocytes; miR-155 is then transferred to the same epithelial cells and induces resistance to therapy [10]. The interplay between malignant cells and the microenvironment is more complicated than initially imagined, and further captivating findings will surely follow.

## Abbreviations

miRNA: MicroRNA
